# Chemopreventive targeted treatment of head and neck precancer by Wee1 inhibition

**DOI:** 10.1038/s41598-020-58509-2

**Published:** 2020-02-11

**Authors:** Anne M. van Harten, D. Vicky de Boer, Sanne R. Martens-de Kemp, Marijke Buijze, Sonja H. Ganzevles, Keith D. Hunter, C. René Leemans, Victor W. van Beusechem, Rob M. F. Wolthuis, Renée X. de Menezes, Ruud H. Brakenhoff

**Affiliations:** 1 Amsterdam UMC, Vrije Universiteit Amsterdam, Otolaryngology/Head and Neck Surgery, section Tumor Biology, Cancer Center Amsterdam, Amsterdam, The Netherlands; 2 Academic Unit of Oral and Maxillofacial Medicine, Surgery and Pathology, University of Sheffield, South Yorkshire, England; 3 Amsterdam UMC, Vrije Universiteit Amsterdam, Medical Oncology, Cancer Center Amsterdam, Amsterdam, The Netherlands; 4 Amsterdam UMC, Vrije Universiteit Amsterdam, Clinical Genetics, section Oncogenetics, Cancer Center Amsterdam, Amsterdam, The Netherlands; 5 Amsterdam UMC, Vrije Universiteit Amsterdam, Epidemiology and Biostatistics, Cancer Center Amsterdam, Amsterdam, The Netherlands

**Keywords:** Targeted therapies, Targeted therapies, Head and neck cancer

## Abstract

HPV-negative head and neck squamous cell carcinomas (HNSCCs) develop in precancerous changes in the mucosal lining of the upper-aerodigestive tract. These precancerous cells contain cancer-associated genomic changes and cause primary tumors and local relapses. Therapeutic strategies to eradicate these precancerous cells are very limited. Using functional genomic screens, we identified the therapeutic vulnerabilities of premalignant mucosal cells, which are shared with fully malignant HNSCC cells. We screened 319 previously identified tumor-lethal siRNAs on a panel of cancer and precancerous cell lines as well as primary fibroblasts. In total we identified 147 tumor-essential genes including 34 druggable candidates. Of these 34, 13 were also essential in premalignant cells. We investigated the variable molecular basis of the vulnerabilities in tumor and premalignant cell lines and found indications of collateral lethality. Wee1-like kinase (*WEE1*) was amongst the most promising targets for both tumor and precancerous cells. All four precancerous cell lines were highly sensitive to Wee1 inhibition by Adavosertib (AZD1775), while primary keratinocytes tolerated this inhibitor. Wee1 inhibition caused induction of DNA damage during S-phase followed by mitotic failure in (pre)cancer cells. In conclusion, we uncovered Wee1 inhibition as a promising chemopreventive strategy for precancerous cells, with comparable responses as fully transformed HNSCC cells.

## Introduction

Head and neck squamous cell carcinomas (HNSCCs) arise in the mucosal lining of the upper-aerodigestive tract and account for around 5% of the total cancer incidence^1,2^. The main risk factors for head and neck cancer are smoking and excessive alcohol consumption, infection with a high-risk type of the human papillomavirus (HPV), or genetic predisposition such as Fanconi anemia (FA)^2^. FA is characterized by congenital abnormalities, progressing anemia, and cancer predisposition, particularly of oral cancers. These tumors are difficult to manage as FA-patients are sensitive to cross-linking agents such as cisplatin, hampering clinical management of tumors, and for these patients surveillance and prevention is key.

Tumors in the head and neck region develop in premalignant mucosal changes, large epithelial areas characterized by cancer-associated genetic changes, also referred to as “fields”. These precancerous fields can be centimeters in size, and are often macroscopically invisible. A minority manifests as visible mucosal lesions known as leukoplakia or erythroplakia, which occur with a prevalence of around 0.1–0.2% for leukoplakia^3^, and 0.02–0.2% for erythroplakia^4^. Every year, 1–2% of the leukoplakia lesions progress into cancer, and erythroplakia lesions inevitably progress.

Despite the occurrence and prevalence of these visible lesions, the large majority of head and neck cancers develop *de novo*. In resected tumor specimen, however, preceding premalignant changes can be often identified in the surgical margins by either microscopic detection of dysplasia or by genetic analysis^5^. When these fields are incompletely removed after tumor excision, they often lead to a local relapse^2,6^. Moreover, independent fields may exist that did not undergo malignant transformation yet, and may cause second primary tumors^7^.

These premalignant fields are difficult to manage clinically. Small visible lesions can be excised or treated using laser. Unfortunately, efficacy is limited as lesions frequently recur and subsequently undergo malignant transformation, or tumors may develop elsewhere. Larger lesions and fields that are not visible cannot be treated yet^2–4^. When tumors occur, treatment is obviously focused on the index tumor, which may comprise of surgical resection with or without post-operative (platinum-based chemo-) radiotherapy, or upfront chemo-radiotherapy^2,8^. Despite these invasive treatment protocols, local relapses frequently occur even when the surgical margins are histologically tumor-free, and often develop from fields that stayed behind unnoticed^2^. Improvement of therapy protocols is therefore necessary to treat not only the tumors more effectively, but also these premalignant fields. Targeted treatment approaches seem an attractive option, however, targetable mutations in oncogenes rarely occur in head and neck (pre)cancer, hampering the identification of suitable drug targets^5,9^. A second problem in designing novel targeted chemopreventive strategies is the lack of *in vivo* models. *In vitro* premalignant oral cell models have been developed, but these do not form detectable premalignancies in *in vivo* xenograft models, complicating preclinical investigations^5^.

Exploration of both synthetic and collateral lethality may form alternative strategies for targeted therapy approaches. Cells that harbor mutations in tumor suppressor genes may become vulnerable for functional losses of other genes or pathways. As an example, frequent inactivation of *CDKN2A* and over-expression of D type cyclins point towards cell cycle aberrations that might cause replication stress and genomic instability, and provide an entry point for targeting strategies through synthetic lethality. Alternatively, HNSCC cells are characterized by frequent chromosomal aberrations that result loss of chromosomal loci associated with inactivation of tumor suppressor genes^9^. With the loss of a locus containing a tumor suppressor gene, neighboring genes are often affected as well, which causes homozygous or heterozygous deletions of these passenger genes^10^. Loss of some of these passenger genes may cause sensitivity to inhibition by siRNAs or drugs, or the cell becomes fully dependent on the paralogue of the (partially) lost gene. These vulnerabilities are named collateral lethality, and these genes can be explored as therapeutic targets^10^.

To investigate new therapeutic approaches to target the invasive cancers, we previously performed genome-wide RNA interference (RNAi) screens^11^, and a panel of over 300 tumor-lethal siRNAs were identified. In the current study, we used a custom library of these lethal siRNAs to further investigate the vulnerabilities of both cancer and premalignant cells compared to normal primary cells.

## Results

### Identification of essential genes

**Figure 1:**
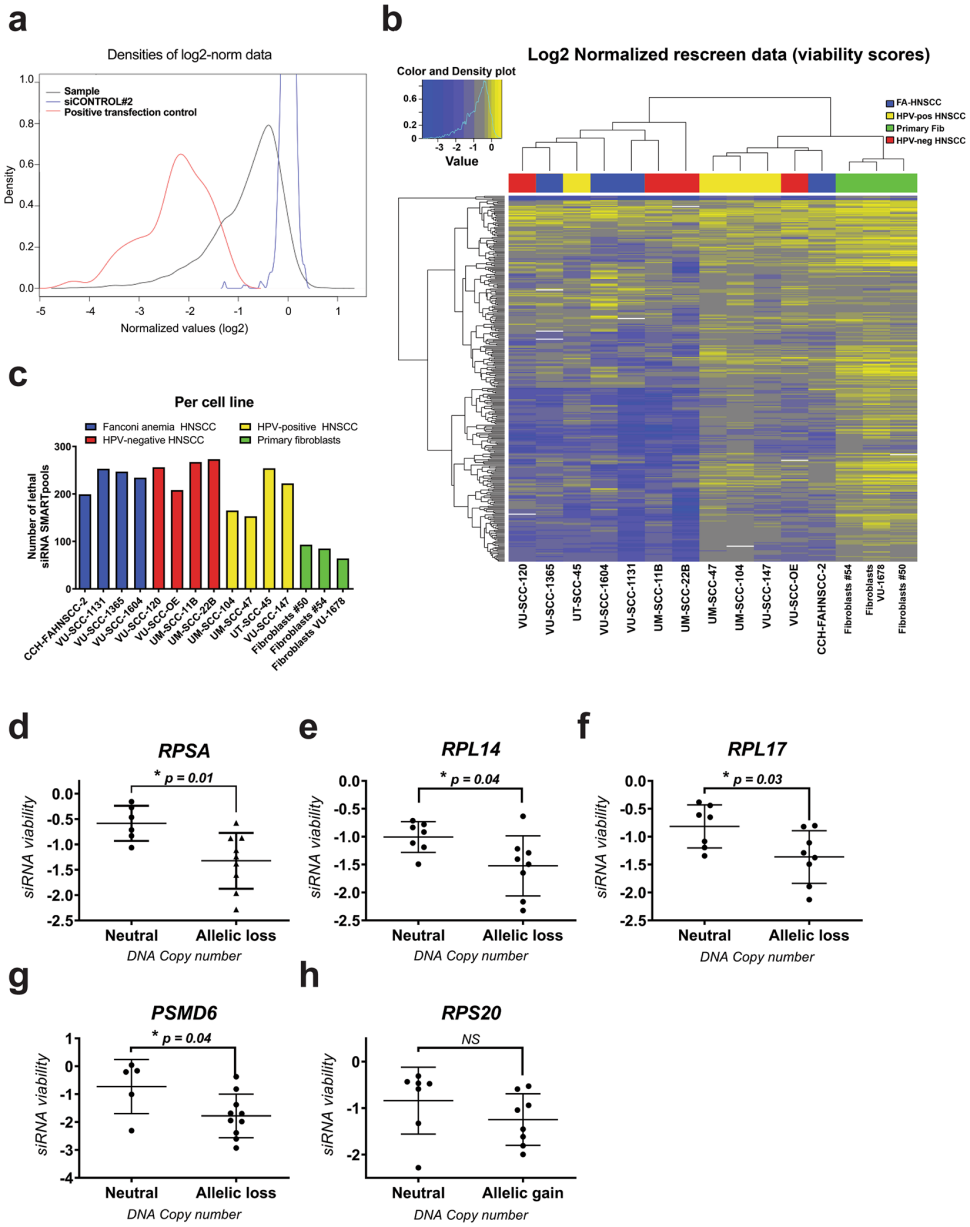
Screening of 319 tumor-lethal siRNAs on a panel of cancer, premalignant and normal primary cell cultures and the exploitation of collateral lethality. (**a)** Distribution of all siRNAs is shown. The x-axis represents the viability score (normalized log2 transformed values). The y-axis represents the density score of the siRNAs. In red, all positive controls (si*UBB*) are shown, and in blue all negative controls (siCONTROL#2) are depicted. An accurate separation between the controls was obtained. The black line represents all 319 siRNAs, which were considered lethal at the viability score cut-off ≤−0.5. (**b)** Cluster analysis of the twelve re-screened HNSCC cell lines. The HPV-negative HNSCC lines are presented in red, in blue the HNSCC lines from Fanconi anemia patients, in yellow the HPV-positive HNSCC lines, and in green three non-transformed fibroblast cultures. The cluster analysis revealed no differences between the cancer sub-groups. Most obvious are the low numbers of hits in the normal primary cells. (**c)** Number of lethal siRNA SMARTpools with a viability score of ≤ −0.5, per cell line. In all three non-transformed oral fibroblasts, less than 100 essential genes were found. (**d–h)** All screened cell lines were divided on the basis of genomic loss analysis in two groups; either the cell line was CN neutral, or had a heterozygous CN alteration of the bins where *RPSA* (**d**), *RPL14* (**e**), *RPL17* (**f**), *PSMD6* (**g**) or *RPS20* (**h**) are located. The group without aberrations displayed significant less reduction in cell viability upon knockdown of *RPSA* (two-sided t-test, p = 0.01), *RPL14* (two-sided t-test, p = 0.04), *RPL17* (two-sided t-test, p = 0.03) and PSMD6 (two-sided t-test, p = 0.04). For *RPS20*, no significant correlation was found between CN status (allelic gain) and siRNA viability.

We constructed a custom siRNA SMARTpool library (Fig. S1a) based on hit selection in previously performed array-based genome-wide siRNA screens in two tumor cell lines. The library consisted of 319 siRNAs targeting genes that were found to be essential in these initial two tumor cell lines^11^. Rescreening of the custom library in the originally screened HNSCC cell line revealed confirmation of 85% of the hits^12^, indicating the accuracy of the approach and these data were also included in this study as a reference ^12^. Here, we extended the cell line panel with three HPV-negative and four HPV-positive HNSCC cell lines, and in addition four HPV-negative HNSCC cell lines established from head and neck tumors in Fanconi anemia (FA-)patients. We further included primary non-transformed oral fibroblasts of two healthy donors and one FA-patient, to identify tumor-specific lethality (Table 1, Fig. S1b). Normalized Log2 transformed data points demonstrated an accurate separation of the positive (e.g. si*UBB*, Ubiquitin B, in red) and negative controls (siCONTROL#2, in blue) (Fig. 1a). As expected the majority of the pre-selected siRNAs (in black) had a lethal effect in most cell lines. To identify the siRNA SMARTpools that induced a relevant decrease in cell viability, median values per cell line were calculated and the cut-off for selection was set at a viability score of −0.5. Using this cut-off >99% of the negative controls were scored as negative and all positive controls were scored as positive.

**Table 1:** Overview of cells screened with the tumor-lethal library.

Cell line	Type	Site	Doubling time (h)	Method of transfection	Transfection reagent	Cells per well	Volume reagent well (μL)
Fibroblasts #50	Primary fibroblasts	Uvula	32	Reverse	RNAiMax	2000	0.05
Fibroblasts #54	Primary fibroblasts	Uvula	20	Reverse	RNAiMax	2000	0.06
Fibroblasts VU-1678	Primary fibroblasts	—	47	Reverse	RNAiMax	2000	0.05
VU-SCC-120 (*reference*)	HPV-negative tumor	Tongue	20	Forward	DharmaFECT #1	1000	0.03
VU-SCC-OE	HPV-negative tumor	Floor of mouth	30	Reverse	DharmaFECT #1	5000	0.08
UM-SCC-11B	HPV-negative tumor	Larynx	22	Forward	DharmaFECT #1	2000	0.035
UM-SCC-22B	HPV-negative tumor	Hypopharynx	23	Forward	DharmaFECT #1	4000	0.1
VU-SCC-1131	FA-derived tumor	Floor of mouth	24	Reverse	RNAiMax	2000	0.02
VU-SCC-1365	FA-derived tumor	Mouth mucosa	27	Reverse	RNAiMax	4000	0.06
CCH-FAHNSCC-2	FA-derived tumor	—	15	Reverse	RNAiMax	3000	0.04
VU-SCC-1604	FA-derived tumor	Tongue	30	Reverse	RNAiMax	2500	0.03
UM-SCC-47	HPV16-positive tumor	Lateral tongue	27	Forward	DharmaFECT #1	5000	0.1
VU-SCC-147	HPV16-positive tumor	Floor of mouth	25	Reverse	DharmaFECT #1	4000	0.1
UT-SCC-45	HPV33-positive tumor	Oral cavity	28	Forward	DharmaFECT #1	2500	0.14
UM-SCC-104	HPV16-positive tumor	Floor of mouth	67	Reverse	DharmaFECT #1	5000	0.11

**-** Unknown.

**FA** Fanconi anemia.

**HPV** Human Papillomavirus.

### Cluster analysis of median viability scores

Unsupervised cluster analysis of the normalized and transformed median cell viability values did not reveal clear differences between the FA patient-derived, HPV-positive and HPV-negative tumor cell lines (Figs. 1b and S1c). Instead, two main clusters were found, which seemed to correspond mainly to the number of hits identified (<200 and >200; Fig. 1c). Within the cluster with <200 essential genes, the primary fibroblasts formed a separate group. The separation into two clusters neither related to differences in transfection reagent, nor to reverse or forward transfection, nor to cell doubling time^12,13^. Next, we assessed the number of identified lethal siRNA SMARTpools per cell line (Fig. 1c). This number ranged from 64 (fibroblast culture) to 273 (UM-SCC-22B) with a median of 222. Most prominent were the low numbers of lethal siRNAs in the primary fibroblasts, less than 100 siRNAs were lethal for all three donors. The difference in lethal hits between primary fibroblasts and tumor cells reveals an exploitable window for selective tumor cell targeting therapies.

### Collateral lethality analysis

The concept of collateral lethality associates genomic aberrations in tumor cells to specific vulnerabilities of gene interference in these cells. We hypothesized that some identified lethal hits within our rescreen could be explained by a heterozygous loss or amplification of the related chromosomal locus. Therefore, we adapted and applied an integrated analysis as previously described by Menezes *et al*.^14^ of the cell line DNA copy number (CN) data per chromosome arm^15^ and the siRNA lethality data per cell line. Aberrations of chromosome arms 3p, 7p, 10p, 8q, 9q and 18q demonstrated significant associations with specific siRNA vulnerabilities of genes located at these loci (Table 2). We identified positive partial correlations between siRNAs and losses at chromosome arms 3p, 10p and 18q (Z-score > 3) and negative partial correlations between siRNAs and gains at chromosome arms 7p, 9q and 8q (Z-score < −3). Associations with gains and amplifications may point to collateral gene addictions, while associations with losses indicates a more classical collateral lethality. Loss of one copy of a tumor suppressor gene by a (partial) chromosomal loss, likely reduces expression of neighboring genes and may increase vulnerability to siRNAs targeting these.

**Table 2:** Collateral lethality.

Gene	Location
*RPSA*	3p22.1
*RPS20*	8q12.1
*RPL14*	3p22.1
*RPL17*	18q21.1
*PSMD6*	3p14.1
*PSMA2*	7p14.1
*SEC61G*	7p11.2
*HNRNPK*	9q21.32
*RPL30*	8q22.2
*RPL8*	8q24.3
*PRPF18*	10p13
*SNRPD1*	18q11.2

Four candidate genes with a positive correlation (*RPSA*, *RPL14*, *RPL17* and *PSMD6*) and one with a negative correlation (*RPS20*) were further investigated by knockdown of expression of these genes with the relevant siRNA SMARTpool. All these genes, except proteasome subunit *PSMD6*, encode for ribosomal proteins. CN data of these specific genes revealed that the viability after transfection indeed positively correlated to losses for *RPSA*, *RPL14*, *RPL17* and *PSMD6*, with p-values of 0.01, 0.04, 0.03 and 0.04, respectively (Fig. 1d–h). These data indicate that collateral lethality by targeting neighboring genes could indeed explain a number of tumor-specific hits and seems to form a genuine basis for hit selection.

### Hit selection in cancer and precancer cell lines

The knockdown effect of many genes was tumor cell line-specific, indicating a large variation in vulnerabilities and limiting applicability of potential inhibitors as a panHNSCC treatment strategy. We therefore mined our rescreen siRNA data for shared vulnerabilities across multiple tumor cell lines (Table S2). Our criterion of a panHNSCC-lethal hit was arbitrarily chosen as a viability score ≤ −0.5 in at least 24 out 36 tumor cell line replicates. Additionally, genes were classified as ‘core fitness’ genes^16^ in both tumor cells and normal cells, when knockdown was also lethal in more than 6 out 9 fibroblast replicates. In total, 197 out of 319 siRNA SMARTpools met the criterion ‘panHNSCC-lethal’, whereas 50 of these siRNA SMARTpools were also lethal in six or more fibroblast replicates. This resulted in 147 panHNSCC target genes and 50 core fitness genes (Table S2).

KEGG pathway analysis using DAVID revealed that the 147 tumor-specific siRNAs can be classified in seven functional subgroups, whereas the 50 core essential genes were mainly ribosomal (Fig. S2a,b)^17^. For further selection of the 147 hits we mined a ‘drug to gene-interaction’ database and this revealed 34 druggable genes (Table S3)^18^.

To further select candidate siRNA SMARTpools that target premalignant cells as well, we interrogated and re-analyzed the data of the previously screened precancerous cell line VU-preSCC-M3 with the same 319 siRNAs^12^. This revealed that 22 genes out of the 34 druggable essential genes in cancer cells are also essential in precancerous cells (Table S3). The top five lethal siRNAs in VU-preSCC-M3 were directed against *KIF11*, *RRM1*, *NHP2L1*, *WEE1* and *SF3B1*, and all showed effect in at least 9 of 12 tumor cell lines (Table S3). *KIF11* encodes for a mitotic spindle protein and was previously identified to be tumor-lethal in HNSCC^11^. *RRM1* is involved in deoxynucleotide synthesis and cell cycle progression. It is also a cellular target for a chemotherapeutic agent, gemcitabine. Interestingly, *NPH2L1* and *SF3B1* are splice factors and both cancer and precancerous cells displayed an increased dependency on splicing^19^.

The most promising hit for clinical implication to target premalignant squamous cells seemed Wee1-like kinase (*WEE1*), a key component in cell cycle control that regulates the activities of cyclin bound CDK1 and CDK2. The Wee1 inhibitor Adavosertib (formerly known as MK-1775 and AZD1775) is currently under investigation in clinical trials to treat solid tumors and seems promising for ovarian and head and neck cancer^20,21^. We therefore investigated the potential of Wee1 inhibition as chemopreventive strategy for mucosal precancer, included HNSCC tumor cell lines, and also related the findings to ovarian cancer cells as reference.

### *WEE1* as druggable target in (pre)malignant cells

All tumor cell lines showed a decreased cell viability with a value ≤ −0.5 upon *WEE1* knockdown, except VU-SCC-1604 (Table S3). Primary oral fibroblasts also responded slightly to *WEE1* knockdown, but did not reach the cut-off.

**Figure 2:**
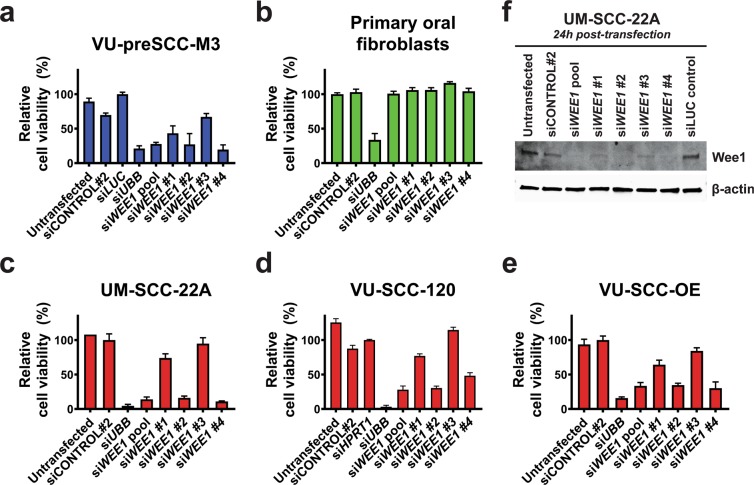
siRNA validations of si*WEE1*. (**a–e)** Deconvolution of the SMARTpool in the four separate siRNAs all targeting *WEE1* in (**a**) VU-preSCC-M3 (precancerous cell line in blue), (**b**) the primary oral fibroblasts (in green), (**c**) UM-SCC-22A, (**d**) VU-SCC-OE, and (**e**) VU-SCC-120 (all HPV-negative HNSCC lines in red). All graphs show the effect on cell viability upon knockdown compared to the negative transfection control used. Negative transfection control siCONTROL#2 was used for primary fibroblasts, UM-SCC-22A, and VU-SCC-OE. Since toxicity was observed with siCONTROL#2 transfection in VU-preSCC-M3 and VU-SCC-120, si*LUC* and si*HPRT1* were used, respectively, which were set at 100% cell viability. (**f)** The Wee1 protein levels were examined on Western blot and revealed a near complete knockdown for the si*WEE1* SMARTpool and si*WEE1* #2 and #4. si*WEE1* #1 and #3 showed a reduced level of Wee1 protein expression 24 h post-transfection. Knockdown effects on Wee1 protein level correlated with the viability effects as found in Fig. 2c. Negative transfection controls siCONTROL#2 and si*LUC* were taken along for comparison with untransfected cells.

We next deconvoluted the si*WEE1* SMARTpool in several cell lines to confirm the re-screening results (Fig. 2a–e). Two out of four individual siRNAs had similar effects on the viability of VU-preSCC-M3 and HNSCC cell lines as the SMARTpool. In contrast, they did not affect the viability of primary oral fibroblasts, indicating a therapeutic window in line with the results from the siRNA re-screen. A near complete Wee1 protein knockdown was observed for the pooled siRNAs and si*WEE1* #2 and #4 24 h post-transfection, whereas knockdown with si*WEE1* #1 and #3 resulted in a slightly reduced protein level (Fig. 2f), an observation concordant with the siRNA transfection effects on cell viability (Fig. 2c).

**Figure 3:**
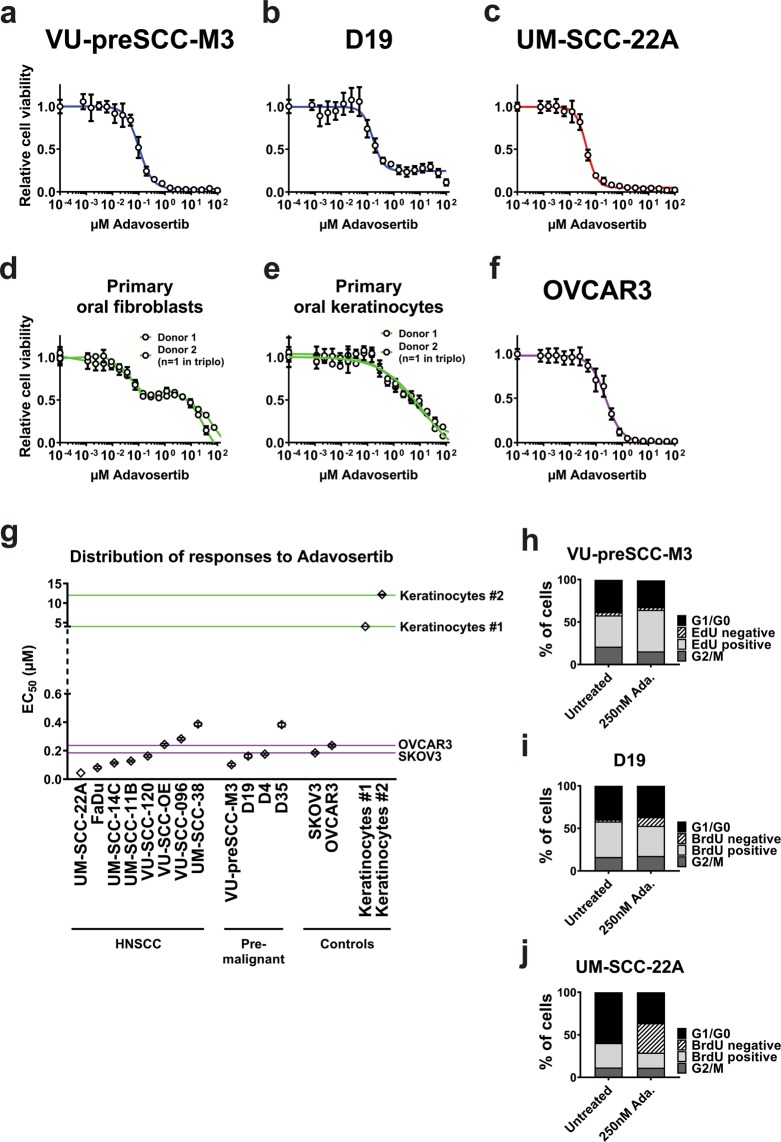
Effects on cell viability and cell cycle distribution upon Wee1 inhibition with the clinically relevant inhibitor Adavosertib. (**a–f**) Line graphs representing the dose responses upon 72 h Adavosertib treatment are shown for premalignant cells (VU-preSCC-M3 (**a**) and D19 (**b**), in blue), HPV-negative HNSCC cell line UM-SCC-22A (**c**, in red), primary oral fibroblasts and keratinocytes (**d**,**e**, both in green) and ovarium cancer cell line OVCAR3 as reference (**f**, in purple). All graphs display the relative cell viability compared to untreated cells upon increasing concentrations of the inhibitor after 72 h of exposure. Relative cell viability to untreated cells is shown for a serial dilution of Adavosertib (at logarithmic x-axis), and the viability of the untreated cells (presented as relative cell viability of 1) is imputed at 100 pM. A larger cell line panel is presented in Fig. S2c–e. (**g)** Overview of the EC_50_-values (µM) of all cell lines tested, except the fibroblasts. The purple lines represent the reference ovarian tumor cell lines and the green lines the EC_50_-values of the primary keratinocyte cultures. The majority of HNSCC and premalignant cells are equally or even more vulnerable to Adavosertib compared to both ovarian tumor lines tested. (**h–j)** Cell cycle distribution of precancerous cell lines VU-preSCC-M3 (**h**) and D19 (**i**), and HNSCC cell line UM-SCC-22A (**j**), after 24 h treatment with Adavosertib. VU-preSCC-M3 was pulsed with EdU for 15 min and subsequently the DNA content was stained with DAPI using the Click-IT method. All other cell lines (including those shown in Fig. S3e) were pulsed with BrdU for 15 min and DNA content was measured using PI. Both precancerous and tumor cells showed aberrant cell cycle distributions.

To further validate the potential of Wee1 inhibition as a chemopreventive therapy, a panel of premalignant cell cultures was treated with Adavosertib, together with a panel of HNSCC and two ovarian tumor cell lines as reference, since ovarian cancer is known to be vulnerable to Wee1 inhibition in clinical studies^20,22^. The tested cell lines all harbored a *TP53* mutation (Table S4). Primary non-transformed oral fibroblasts and keratinocytes from healthy donors served as toxicity controls to indicate a potential therapeutic window compared to premalignant and tumor cells. All HNSCC tumor cell lines and premalignant cells responded in comparable concentration ranges as the tested ovarian tumor cell lines OVCAR3 and SKOV3, or were even more sensitive (Figs. 3a–g and S2c–e). The tested primary oral cells did not respond to Wee1 inhibition, or at least at much higher concentrations. Primary fibroblasts from two independent donors showed a remarkable biphasic response, which may suggest inhibition of a secondary target of Adavosertib affecting fibroblasts specifically. We could not determine the EC_50_-values of the fibroblasts due to this biphasic response. The tested primary keratinocytes did not show a biphasic response and the EC_50_-values were 10 to 100 times higher than the EC_50_-value of cell line UM-SCC-38 (highest EC_50_) or UM-SCC-22A (lowest EC_50_), respectively (Fig. 3g). The therapeutic window between the primary keratinocytes on the one hand, and the squamous cancer and precancerous cells on the other, as well as the comparable responses in comparison to ovarian tumor cell lines are promising for clinical applications of Adavosertib in HNSCC and oral precancer.

Combination of Wee1 inhibition with γ-irradiation or PARP inhibition have been under investigation for other cancer types^23–25^. Despite reports of additive or synergistic effects in other tumor cell models, combining Wee1 inhibition with irradiation did not reveal an additional therapeutic effect for HNSCC cell lines. Also the combination of Adavosertib with the highly specific PARP catalytic inhibitor and potent trapper of PARP1 Talazoparib did not enhance therapeutic efficacy in HNSCC-derived cell lines (Fig. S3a,b).

Besides Wee1 and CDC25, Chk1 plays a key role in cell cycle control and regulation of CDK1 activity, and we wondered whether responses to Chk1 inhibitors correlate to those of Wee1 inhibitors. Although the cell line panel is small, no correlation was found between sensitivities to Chk1 inhibition with Rabusertib (LY2603618)^26^ and Wee1 inhibition (Fig. S3c; n = 6, Pearson’s correlation R = −0.04, p = 0.94), implying distinct regulatory mechanisms. Furthermore, no correlation was found between cell line sensitivity to cisplatin treatment^13^ and Wee1 inhibition (Fig. S3d; n = 8, Pearson’s correlation R = 0.63, p = 0.10), which suggests that patients who do not respond to classical chemo-radiation with cisplatin might be well treatable by Wee1 inhibitors (Fig. 3g).

### Wee1 inhibition and cell cycle progression

In HNSCC cell cycle control is frequently abrogated at G1/S by somatic changes in *CDKN2A*, cyclin D1 and *TP53*^8^. Premature S-phase entry causes replication stress and demands more precise S-phase control by Chk1^26^ and control at G2/M. The complex of CDK1-Cyclin B is the key regulator of G2-to-M transition and onset of mitosis, and regulates to a lesser extent S-to-G2 transition^27^. Wee1 regulates CDK1 activity through Y15-phosphorylation, thereby preventing premature CDK1 activation and mitotic entry. As most premalignant cells also have genetic changes in *TP53* and *CDKN2A*^5^, we questioned whether precancerous cells face comparable problems in replication and cell cycle regulation as HNSCC cells, and respond comparably to Wee1 inhibition. Therefore, we investigated cell cycle progression after 24 h of Wee1 inhibition (Figs. 3h–j and S3e). Both premalignant cell lines VU-preSCC-M3 and D19 showed an increased S-phase population after 24 h of Wee1 inhibition, although VU-preSCC-M3 had an increase in replicating S-phase (EdU-positive cells) and D19 in non-replicating S-phase (BrdU-negative cells). In fact, all tested HNSCC and OVCAR cell lines, except UM-SCC-22A and UM-SCC-14C, showed an increased population of non-replicating S-phase cells at baseline when compared to the premalignant cells, implying a more severe cell cycle disruption at baseline. After treatment, a majority of cell lines in this panel showed an S-phase increase (D19, VU-preSCC-M3, UM-SCC-22A, UM-SCC-14C, UM-SCC-11B, VU-SCC-120, UM-SCC-38 and SKOV3), while two lines displayed a G1 increase (VU-SCC-096 and OVCAR3), which seemed to relate to sensitivity to Adavosertib as the latter two are less sensitive.

### Wee1 inhibition initiates death in mitosis and micro-nucleation due to DNA damage induction and unsupervised entry of mitosis

**Figure 4:**
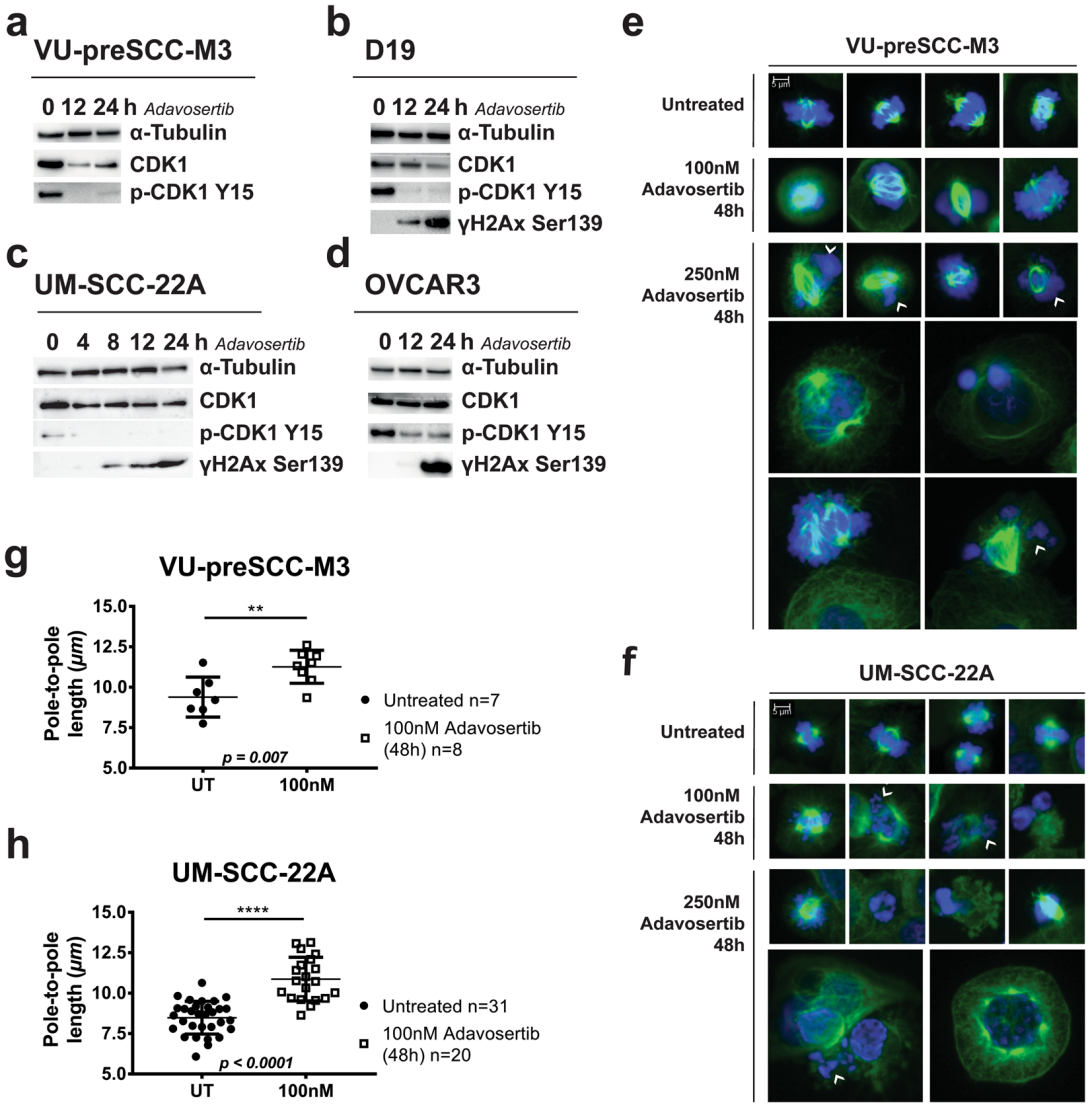
Effects of Wee1 inhibition on cell cycle regulation, DNA damage and mitosis. (**a–d)** Western blot analysis of premalignant cell lines VU-preSCC-M3 (**a**) and D19 (**b**), HNSCC cell line UM-SCC-22A (**c**) and ovarian cell line OVCAR3 (**d**) all showed decreased p-CDK1 Y15 expression upon treatment with 100 nM Adavosertib, already after 4 h of treatment (**c**). Increased levels of γH2Ax Ser139 indicated DNA damage induced by Wee1 inhibition. Additional cell lines are shown in Fig. S4a–c. (**e,f)** Precancerous cells VU-preSCC-M3 (**e**) and HNSCC UM-SCC-22A (**f**) were stained for α-tubulin (microtubules, in green) and DNA (DAPI, in blue). In untreated conditions, both the premalignant and tumor cell lines showed normal bipolar spindle formation with condensated chromosomes. Upon treatment with 100 nM Adavosertib for 48 h, (pro-)metaphase cells showed less dense spindles with problematic DNA alignment, as well as tripolar spindles and micronuclei formation in UM-SCC-22A. After treatment with 250 nM Adavosertib, chromosomal alignment and condensation was increasingly abnormal in both cell lines and giant cells with multiple nuclei or nuclei fragmentation were found, a result of mitotic failure. No monopolar spindles were found. (**g,h**) To investigate mitotic exhaustion due to SAC checkpoint activation, pole-to-pole length (µm) was measured of (pro-)metaphases in untreated and 100 nM Adavosertib treated cells. A significant increase of pole-to-pole length was found in both VU-preSCC-M3 (**g**, two-sided t-test, p = 0.007) and UM-SCC-22A (**h**, two-sided t-test, p = 0.0001) (see Fig, S4d for a representative picture).

Since Wee1 is a main inhibitor of CDK1, which controls mitosis, the molecular activation of a number of key regulators of mitosis was further investigated (Figs. 4a–d and S4a–c). Expression levels of CDK1 and its binding partner cyclin B1 (not shown) remained more or less stable upon Wee1 inhibition, although the large number of dying cells after 24 h of treatment might have hampered interpretation of the data. Upon treatment with 100 nM Adavosertib, the inhibitory phosphorylation of Y15 on CDK1 decreased, confirming effective Wee1 inhibition. Furthermore, induction of DNA damage marker γH2Ax Ser139 was observed in both premalignant cells and tumor cell lines after Adavosertib exposure, indicating that the loss of Wee1 activity impacts replication stress and associated DNA damage, or impairs DNA repair signaling. Taken together, cancer cells that are in S-phase or in G2-phase, enter mitosis prematurely by Wee1 inhibition, which leads to mitotic failure, in line with the cell cycle distribution upon Wee1 inhibition.

Next, we investigated abnormalities in mitotic cells in precancerous VU-preSCC-M3 and HNSCC UM-SCC-22A cells after 48 h of Wee1 inhibition. Microtubules and spindle formation were visualized using α-Tubulin (green) and DNA was stained with DAPI (blue) (Fig. 4e,f). In untreated conditions, both premalignant as well as HNSCC cells showed normal bipolar spindles and chromosomal condensation during mitosis. After Wee1 inhibition, the spindles showed abnormalities and the chromosomes failed to align in the metaphase plate. Both giant cells with fragmented DNA (arrows) and isolated DNA nuclei appeared frequently, indicating mitotic failure. Remarkably, upon treatment with 100 nM Adavosertib, we observed a significant increase in the spindle pole-to-pole distance in (pro-)metaphase cells, compared to untreated cells for both VU-preSCC-M3 (two-sided t-test, p = 0.007) and UM-SCC-22A (two-sided t-test, p < 0.0001) (Figs. 4g,h and S4d). This indicates mitotic arrest due to SAC-checkpoint activation, inward forces on the spindles due to DNA damage caused by Wee1 inhibition, and spindle exhaustion^28–30^. The treatment condition with 250 nM Adavosertib was already too toxic and did not contain enough (pro-)metaphases for further analysis.

## Discussion

We re-screened a previously identified panel of siRNA SMARTpools that target essential genes in two cancer cell lines. We could confirm 62% of the previously identified genes as essential in a large cell line panel of different HNSCC subclasses, obtaining insight in possible new targets for therapy of precancerous cells, as well as HPV-negative, HPV-positive and FA-derived tumors^11^. Of the confirmed lethal siRNAs, 25% target core essential genes of which knockdown is also lethal for primary oral fibroblasts^16^, but 75% seem cancer cell-specific indicating the opportunities for tumor-specific targeting. However, broad application is complicated by the observation that responses varied considerably between cell lines. Although this likely reflects the inter-tumoral heterogeneity of HNSCC^31^, it demands clinical development of inhibitors in parallel with companion diagnostic biomarkers for patient selection^9^.

Numerous novel candidate cancer genes have been identified by the sequencing of over 500 HNSCCs by the TCGA consortium, which again stresses the inter-tumoral heterogeneity of the disease despite that it develops from just one cell type in one tissue^9^. Disappointingly, the sequencing efforts also demonstrated the lack of druggable oncogenic mutations. Nonetheless, the many mutated genes as well as the multiple chromosomal aberrations may form a source for druggable targets by synthetic or collateral lethality. We identified many more lethal hits in the screened tumor cell lines than in the primary oral cells. The association of these essential genes with the genetic changes in these tumor cells suggest that collateral lethality does provide a novel strategy to identify vulnerabilities^32^. Upon the loss of a bona fide tumor suppressor gene through genetic aberrations, a neighboring passenger gene is often homozygously or heterozygously lost as well. These neighboring gene losses were considered futile, but in recent studies it has been demonstrated that the genes located at these loci may play important roles in a variety of key metabolic and regulatory functions, and the subsequent decrease in expression can be exploited as treatment strategy^10,33,34^. Further investigation of collateral lethal hits could lead to the identification of future targets for therapy^32^.

Besides options for tumor treatment, we identified gene targets for chemopreventive treatment of premalignant changes. The number of targets for treatment of premalignant cells is relatively low, likely associated with the smaller number of genetic changes^5,12^. Nevertheless, there are promising candidates such as PLK1^12^ and Wee1-like kinase as presented in this study.

Wee1 is an important negative regulator of CDK1, and critically for correct entry into mitosis at the end of G2-phase^35^. In a *TP53* mutated background, Wee1 is thought to be involved in S-phase regulation as well through CDK2  phosporylation^25^. In our data, we noted after Wee1 inhibition a variety of changes in cell cycle control, DNA damage, and abnormal mitosis. This clearly suggests a key role of Wee1 in HNSCC cells, and involvement of Wee1 in squamous (pre)cancer cell cycle regulation. Unexpectedly, responses to Adavosertib differed between primary keratinocytes and fibroblasts *in vitro*. It has been reported that Adavosertib targets the Yes tyrosine kinase with 2- to 3-fold less potency^22^. Others reported that Yes knockdown increases proliferation and migration in fibroblasts, potentially explaining the slight increase in viability in the tested fibroblasts^36,37^. Interestingly, we compared the sensitivity to Wee1 inhibition in a *TP53* wild-type HNSCC cell line and after *TP53* CRISPR/Cas9 knockout, and no difference in vulnerability was observed suggesting a somewhat more complex interaction with mutated *TP53*^15^. The available inhibitor Adavosertib, currently used in several clinical trials in combination with other drugs, is reported to be well tolerated, with fatigue, nausea, thrombocytopenia and diarrhea as most common adverse events^20–22,38^. First results as single agent or when used in a combination therapy seem promising, with an overall response rate of 43% when combined with carboplatin in a phase II study in patients with *TP53*-mutated ovarian cancer^20^. In a recent neoadjuvant study in HNSCC patients, initial responses when combined with platinum and 5-FU were also promising^21^.

Here we show that Adavosertib is effective in premalignant cells as well. Hence, Wee1 inhibition monotherapy could well serve as chemopreventive strategy to eradicate precancerous changes and prevent cancer and local relapse. Importantly, premalignant cells were comparably sensitive to Wee1 inhibition as the fully transformed HNSCC cancer cell lines and demonstrated comparable mitotic failures^39–41^. The chemopreventive potential of Wee1 inhibitors could be investigated in patients with an (erythro-)leukoplakia lesion that harbor genetic changes such as 3p and 9p losses^42^. In addition, it could be applied as adjuvant chemopreventive therapy after surgical resection in HNSCC patients with dysplastic and genetic changes in the surgical margins to prevent local recurrence and second primary tumors^5^. In a phase I/II study, an intermediate endpoint could be exploited such as shrinkage of the lesion with photo-documentation or auto-fluorescence, and also by histology and genetic changes in biopsies and brushed samples^43–45^. In a subsequent phase III study, the efficacy in terms of cancer prevention could be evaluated as endpoint.

In conclusion, we show that there is a multitude of druggable targets for squamous cancer and premalignant cells, despite the fact that HNSCC is a tumor suppressor gene driven disease^9^. An excellent and promising drug target for both the tumors and the precancerous fields is Wee1-like kinase, efficiently targeted by Adavosertib. The rewiring of cell cycle control by the frequent inactivation of *TP53* and *CDKN2A* in early carcinogenesis of HNSCC, is an excellent approach for targeted treatment of tumors and precancerous mucosal changes.

## Methods

### Cell culture

Use of residual tissue samples was carried out according to the guidelines of the Dutch Medical Scientific Societies (www.federa.org). The studies with extra biopsies were approved by the Institutional Review Board of Amsterdam UMC, location VUmc and patient specimen were used after written informed consent. Primary cells were collected from excised uvulas of healthy adults as previously described^46^. The fibroblasts were cultured in Dulbecco’s Modified Eagle’s Medium (DMEM, Lonza, BE12-707F), with 10% fetal bovine serum (FBS, Biological Industries, 04-007-1 A) and 2 mmol/L L-glutamine (Lonza, BE17-605F). Primary keratinocytes and VU-preSCC-M3 cells were cultured in serum-free KGM medium (KGM-SFM, Gibco, 17005042), supplemented with 0.1% BSA (MP Biomedicals, 9048-46-8), 25 mg bovine pituitary extract (Gibco, 17005042), 2.5 µg human recombinant EGF (Gibco, 17005042), 250 µg Amphotericin B (Gibco, 15290018) and 250 µg Gentamycin (Sigma-Aldrich, G1397). D4, D19 and D35 were obtained from Prof. K. D. Hunter^47,48^ and cultured in DMEM supplemented with 10% FBS, 2 mmol/L L-Glutamine, 1 × 10^−7^ M Insulin (Sigma, I9278), 2.5 × 10^−4^ M Hydrocortisone (Sigma, H0888) and 2.5 µg/L EGF (Invitrogen, PHG6045).

FaDu, OVCAR3 and SKOV3 were obtained from the American Type Culture Collection. The other used cell lines were obtained and cultured as described before^5,12,13,15,49–51^. All cells were cultured without antibiotics, were mycoplasma negative and checked regularly (Mycoalert, Lonza, LT07-318), and were authenticated by visual inspection and genetic profiling on indication. Cell lines were cultured to a maximum of four months after thawing. The *TP53*-mutation status of all precancerous lines and OVCAR3 and SKOV3 were determined as described before^52^.

### siRNA re-screen and data analysis

A library of 319 lethal siRNA SMARTpools was custom-made based on previous results^11^. Array-based re-screening of this library and read-out of cell viability was performed in a 96-wells plate (Greiner-Bio one, 655180) in triplicate for every cell line as described (Table 1)^12^. The data was Log2 transformed and normalized for replicate and plate effects per cell line using a linear regression model, based on the negative controls present on every plate (siCONTROL#2, Dharmacon, D-001206-14). Median values of the triplicates were calculated to determine the effect on viability, providing a viability score for every siRNA tested.

### Collateral lethality

Low-coverage whole-genome sequencing of the cell lines was performed as described previously^5,15^. The obtained DNA copy number (CN) data and siRNA viability scores were analyzed using the gene-set model approach^14^. Briefly, each CN-variable and viability score of all siRNAs located on the same chromosome arm were associated. This provided a p-value for each CN-variable, subsequently corrected for multiple testing using Benjamini-Hochberg’s FDR method^53^.

### siRNA deconvolution

siRNA deconvolution was performed as described (Table S1)^12^.

### Dose-response curves and combination therapies

24 h after seeding cells in a 96-wells microplate in a pre-determined confluency, the small molecule Wee1 inhibitor Adavosertib (AZD1775/MK-1775, Biovision, 2373) or PARP inhibitor Talazoparib (AvonMedchem, Avon2502) was diluted in a serial dilution of 0.8 nM to 100 µM for tumor cell lines and precancerous cells, and in 0.1 nM to150 µM for primary cells (DMSO < 1%). After 72 h, cell viability was determined using CellTiter-Blue (Promega, G8080) and a GloMax microplate reader (Promega, GM3000). Cells were tested three times in triplicate.

For combination experiments with γ-irradiation, cells were seeded in a 24-wells format at low density, irradiated and treated with the inhibitor 24 h later using a ^60^Co source (Gammacell 220, MDS Nordion). When wells reached 90% confluency at day 9, cells were fixed using 2% paraformaldehyde (Sigma-Aldrich, P6148) for 15 min, and stained for 30 min using crystal violet (Sigma-Aldrich, C0775, 1 mg/mL in H_2_O). After drying, crystal violet absorbance was obtained by adding 10% acetic acid for 20 min and measuring absorbance at λ590 nm.

### Cell cycle analysis

Cells were seeded in a T25 flask and when 50% confluence was reached, treated for 24 h with Adavosertib. Before harvest, cells were pulsed with 4nmol/L 5-bromo-20-deoxyurdine (BrdU, Sigma-Aldrich, 19–160) for 15 min, then harvested and fixed in 75% ice-cold EtOH overnight. Next, cells were incubated with 0.5 mg/mL RNAse A, permeabilized with 5 mol/L HCl:0.5% Triton X-100 for 20 min and HCl neutralized with 0.1 mol/L Na_2_B_4_O_7_. BrdU was stained overnight at 4 °C with mouse-anti-BrdU antibody (clone BU20a, M0744, Dako), and fluorescein isothiocyanate (FITC)-labeled with a conjugated rabbit anti-mouse antibody (F0313, Dako). DNA content was stained with propidiumiodide (PI) (5 µg PI per 10^6^ cells, Sigma-Aldrich, P4170).

For VU-preSCC-M3, the Click-iT™ EdU Alexa Fluor™ 488 Flow Cytometry Assay Kit (ThermoFisher Scientific, C10420) was used according to the protocol of the manufacturer, and DNA was stained with 4′,6-diamidino-2-phenylindole (DAPI).

BD LSR II Fortessa^TM^ and BD FACSDiva^TM^ software V8.0.1 (BD Biosciences) were used for flow cytometry and data analysis.

### Western blot analysis

Whole cell lysates were obtained by RIPA lysis and extraction buffer (Thermo Scientific, 89900) containing 1x HALT™ protease and phosphatase inhibitor EDTA-free cocktail (Thermo Scientific, 78441). Protein content was measured with Pierce^TM^ BCA Protein assay (Thermo Scientific, 23225). Normalized whole cell lysates were run on 4–12% pre-casted gradient SDS-PAGE gels (Bolt Bis-Tris Plus gels, Invitrogen, NW04120) using MOPS or MES running buffer. After blotting with TG buffer (BioRad, 1610771), SuperSignal® West Pico Chemiluminescent Substrate (Thermo Scientific, 34580) was used for development on Uvitec 47 Alliance reader (Uvitec Cambridge) or Odyssey® CLx Imaging System (Li-COR). Antibodies used are listed in Table S1. All original blots can be found in Fig. S5a,b.

### Immunofluorescent staining

Microscopic glass slides were prepared as described previously^11^ and pictures were obtained using a Leica DM6000. Analysis and pole-to-pole measurements were performed using LAS Application Suite X (version 3.4.2, Leica).

### Statistical analysis

All statistical analyses were performed using Graphpad Prism (version 7 and 8) or R (version 3.4.3). The statistical package SIM (version 1.48)^14^ was used for the integrated analysis of the copy number and siRNA viability score.

## Supplementary information

Supplementary information.

Supplementary Table S2.

Supplementary Table S3.
