# Supplementary material for: Chemopreventive targeted treatment of head and neck precancer by Wee1 inhibition
Source: Sci Rep. 2020 Feb 11;10:2330. doi: 10.1038/s41598-020-58509-2 (PMC7012863; doi:10.1038/s41598-020-58509-2)
Supplement: Supplementary file 1 — Supplementary information. [file 41598_2020_58509_MOESM1_ESM.pdf]

Supplementary information

Chemopreventive targeted treatment of head and neck precancer by Wee1 inhibition

Anne M. van Harten[1*], D. Vicky de Boer[1*], Sanne R. Martens-de Kemp[1], Marijke Buijze[1], Sonja H. Ganzevles[1], Keith D. Hunter[2], C. René Leemans[1], Victor W. van Beusechem[3], Rob M. F. Wolthuis[4], Renée X. de Menezes[5], Ruud H. Brakenhoff[1]

** Anne M. van Harten and D. Vicky de Boer contributed equally to this work.*

[1] Amsterdam UMC, Vrije Universiteit Amsterdam, Otolaryngology/Head and Neck Surgery, section Tumor Biology, Cancer Center Amsterdam, The Netherlands
[2] Academic Unit of Oral and Maxillofacial Medicine, Surgery and Pathology, University of Sheffield, South Yorkshire, England
[3] Amsterdam UMC, Vrije Universiteit Amsterdam, Medical Oncology, Cancer Center Amsterdam, The Netherlands
[4] Amsterdam UMC, Vrije Universiteit Amsterdam, Clinical Genetics, section Oncogenetics, Cancer Center Amsterdam, The Netherlands
[5] Amsterdam UMC, Vrije Universiteit Amsterdam, Epidemiology and Biostatistics, Cancer Center Amsterdam, The Netherlands

Corresponding author: Ruud H. Brakenhoff, Amsterdam UMC, Vrije Universiteit, Amsterdam Otolaryngology/Head and Neck Surgery, Tumor Biology section, Cancer Center Amsterdam, PO Box 7057, 1007 MB Amsterdam, The Netherlands. rh.brakenhoff@vumc.nl. Phone: +31-20-4440953.

Keywords: HNSCC, premalignant, Wee1, chemoprevention, collateral lethality

Financial support: The study was supported by the VUmc CCA Foundation (Grant nr.: CCA-2012-2-06), the VUmc Cancer Center Amsterdam and Amsterdam UMC.

Conflicts of interest: The authors disclose no potential conflicts of interest.

Supplementary figures and figure legends

Figure S1: Re-screening of 319 tumor-lethal siRNAs.

a. Overview of the workflow of the re-screen of 319 siRNA SMARTpools, selected from two genome-wide siRNA screens, on an extended cell line panel of twelve tumor cell lines, one precancerous cell line and three primary fibroblast cultures. Next, data analysis and selection revealed 197 lethal siRNAs, of which 147 were tumor-specific. Further analysis revealed 34 druggable genes, of which 22 were essential in the precancerous cells as well. Targeted therapy was explored by collateral lethality as well as validation of one specific target gene (*WEE1*).

b. Graphical presentation of the re-screening of the extended cell line panel with the 319 siRNA SMARTpools. Cells were either forwardly transfected 24h after seeding, or reversely by transfecting cells during seeding. After 96h of siRNA transfection, cell viability was measured using CellTiter-Blue.

c. Mean number of lethal siRNA SMARTpools per group of cell lines. The primary oral fibroblasts showed over three times less lethal hits, compared to the HNSCC cell line groups.

Figure S2: Hit identification and dose-responses of additional HNSCC, precancerous and ovarium cancer cell lines treated with Adavosertib.

a. DAVID analysis showed that the 147 tumor-specific essential genes were classified in seven subgroups: mitosis, helicase, RNA polymerase, translational initiation, proteasome, spliceosome and ribosome.

b. DAVID analysis of the 50 core essential genes of which knockdown was lethal in HNSCC and primary cells were found to be ribosome associated genes.

c-d. The responses to 72h Adavosertib treatment are shown for additional HNSCC cell lines (**c**), precancerous cells (**d**) and an ovarian cancer cell line (**e**), respectively. The relative cell viability to untreated cells is shown for a serial dilution of Adavosertib (at the logarithmic x-axis), and the viability of the untreated cells (represented as relative cell viability of 1) is imputed at 100 pM.

Figure S3: Combination therapy of Adavosertib with PARP inhibitor Talazoparib, γ-irradiation and Chk1 inhibitor Rabusertib.

a. Adavosertib was combined with PARP inhibitor Talazoparib for 72h. No increased sensitivity was observed after combining these treatments in HNSCC cell lines UM-SCC-22A and VU-SCC-120.

b. Combination of γ-irradiation with Adavosertib slightly sensitized HNSCC cell lines VU-SCC-120 and FaDu to Wee1 inhibition.

c. Chk1 and Wee1 are both important regulators of cell cycle progression. No correlation was found between responses to inhibition of Chk1 or Wee1, indicating that sensitivities to those drugs are based on different mechanisms.

d. Cell line sensitivity to cisplatin treatment does not correlate with response to Wee1 inhibition, suggesting that patients not responding to cisplatin treatment might still benefit from Wee1 inhibition.

e. Additional cell cycle distributions of HNSCC and ovarian cancer cell lines are shown in order of EC_{50}-value (Figure 3g). Cell cycle was altered in all tested cell lines, most showing an increased (non-replicating) S- or G2-phase.

Figure S4: Cell cycle deregulation was found upon Adavosertib treatment.

a-c. HNSCC cell lines UM-SCC-11B (**a**), VU-SCC-OE (**b**) and UM-SCC-38 (**c**) all showed decreased p-CDK1 Y15, and increased DNA damage (γH2Ax Ser139) upon Wee1 inhibition. For the loading order of CDK1, p-CDK1 Y15 and α-tubulin of UM-SCC-11B (**a**), the lysates of 12h and 24h treatment were loaded in revered order by mistake for p-CDK1Y Y15, as indicated by the white spaces

between the lanes. For convenience, all original blots are supplemented in Figure S5a-b.

d. Example shown of HNSCC cell line UM-SCC-22A for the pole-to-pole measurements in untreated and Adavosertib treated conditions, obtained with Leica LAS X software.

Figure S5: All uncropped Western blots.

a. Uncropped blot of Wee1 expression 24h post-transfection, as shown in Figure 2f. Blot was imaged at a high intensity using the Odyssey® CLx Imaging System, to ensure that the observed knockdown on protein after siRNA transfection was accurate.

b. All Western blots of Figure 4a-d and S4a-c. The shown uncropped blots were annotated with the corresponding figures as found in the manuscript. All gels were ran with the same input normalized whole cell protein lysate. To enable the detection of multiple proteins per blot, the membrane was cut, as shown in the marker image on top, followed by different exposures that represent the cropped figures. Representable exposure per protein and cell line are annotated using a square. Protein expression of α-tubulin, CDK1, p-CDK1 Y15 and γH2Ax Ser139 is shown per cell line (VU-preSCC-M3, D19, OVCAR3, UM-SCC-22A, UM-SCC-38, UM-SCC-11B, VU-SCC-OE).

Supplementary Figure S1

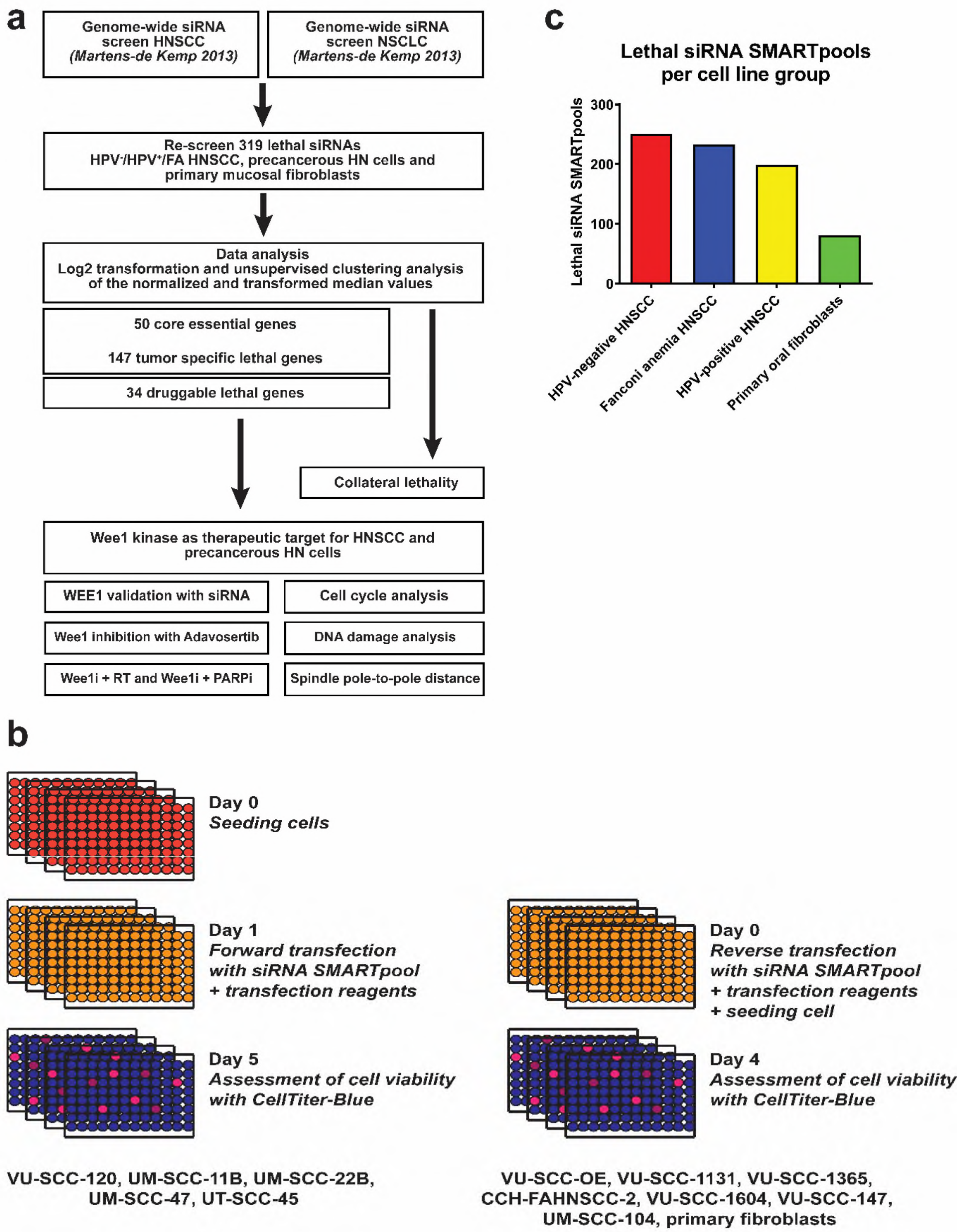

van Harten, de Boer *et al.*

Supplementary Figure S2

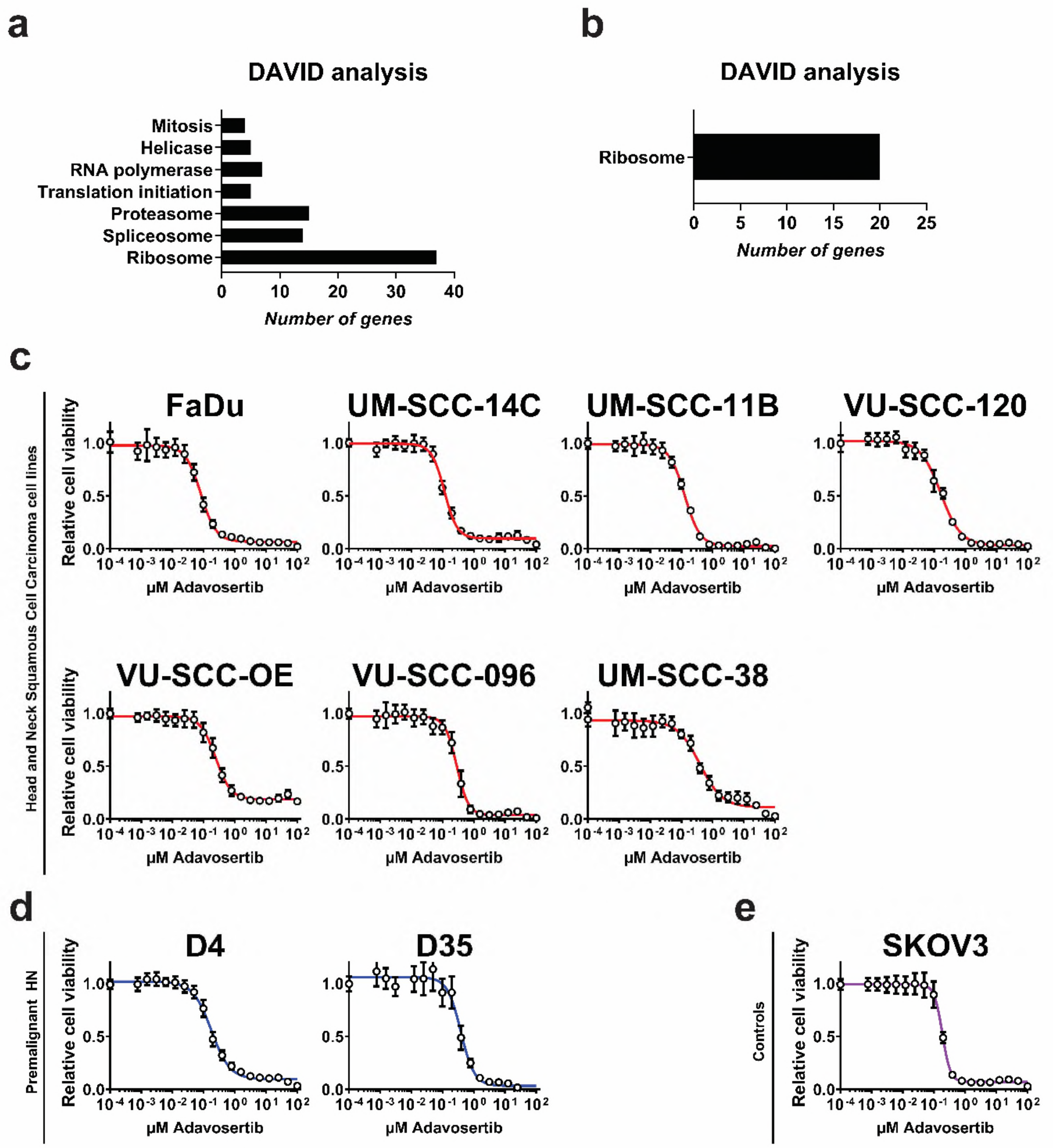

van Harten, de Boer *et al.*

van Harten, de Boer *et al.*

Supplementary Figure S3

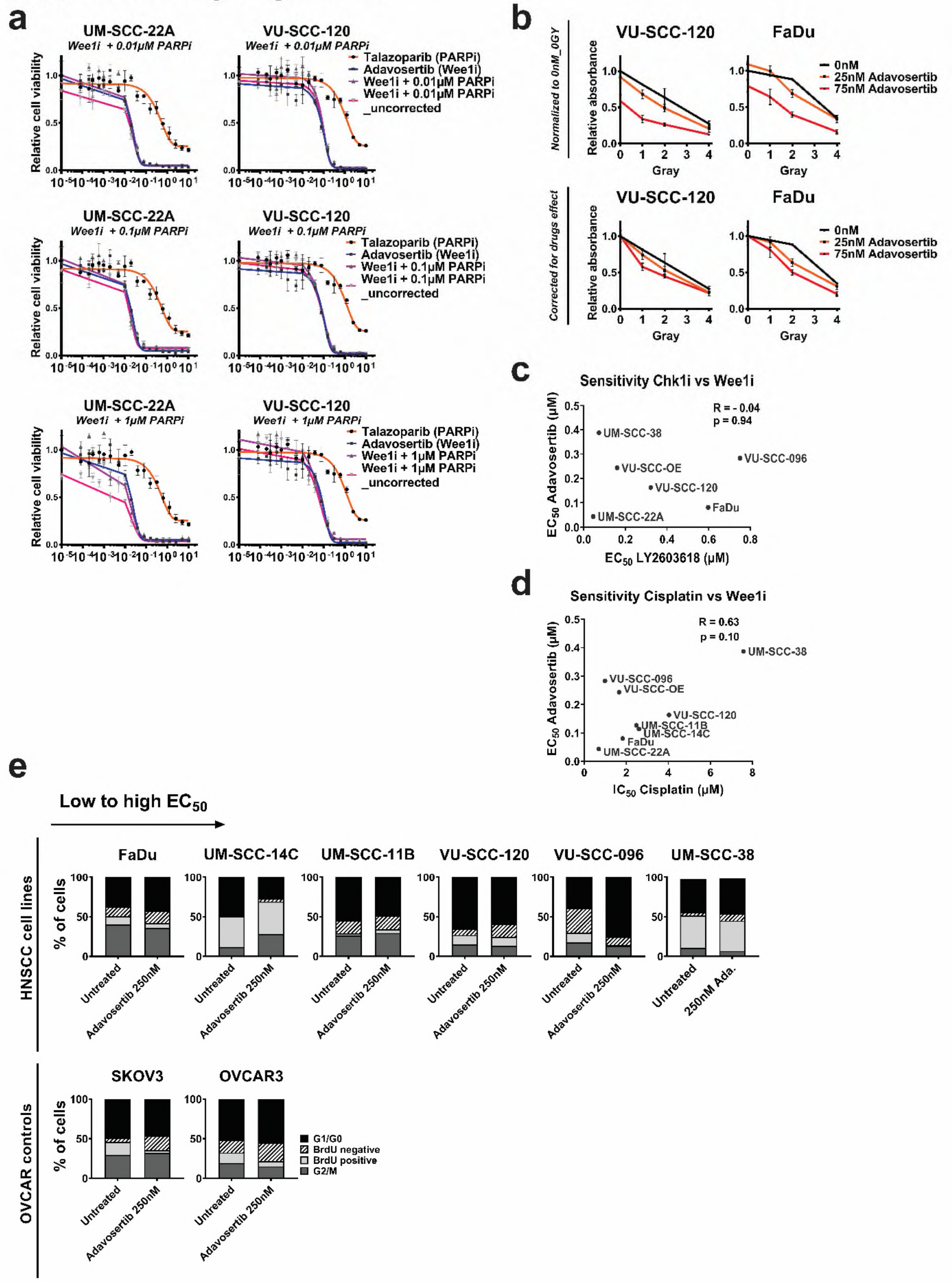

van Harten, de Boer *et al.*

van Harten, de Boer *et al.*

Supplementary Figure S4

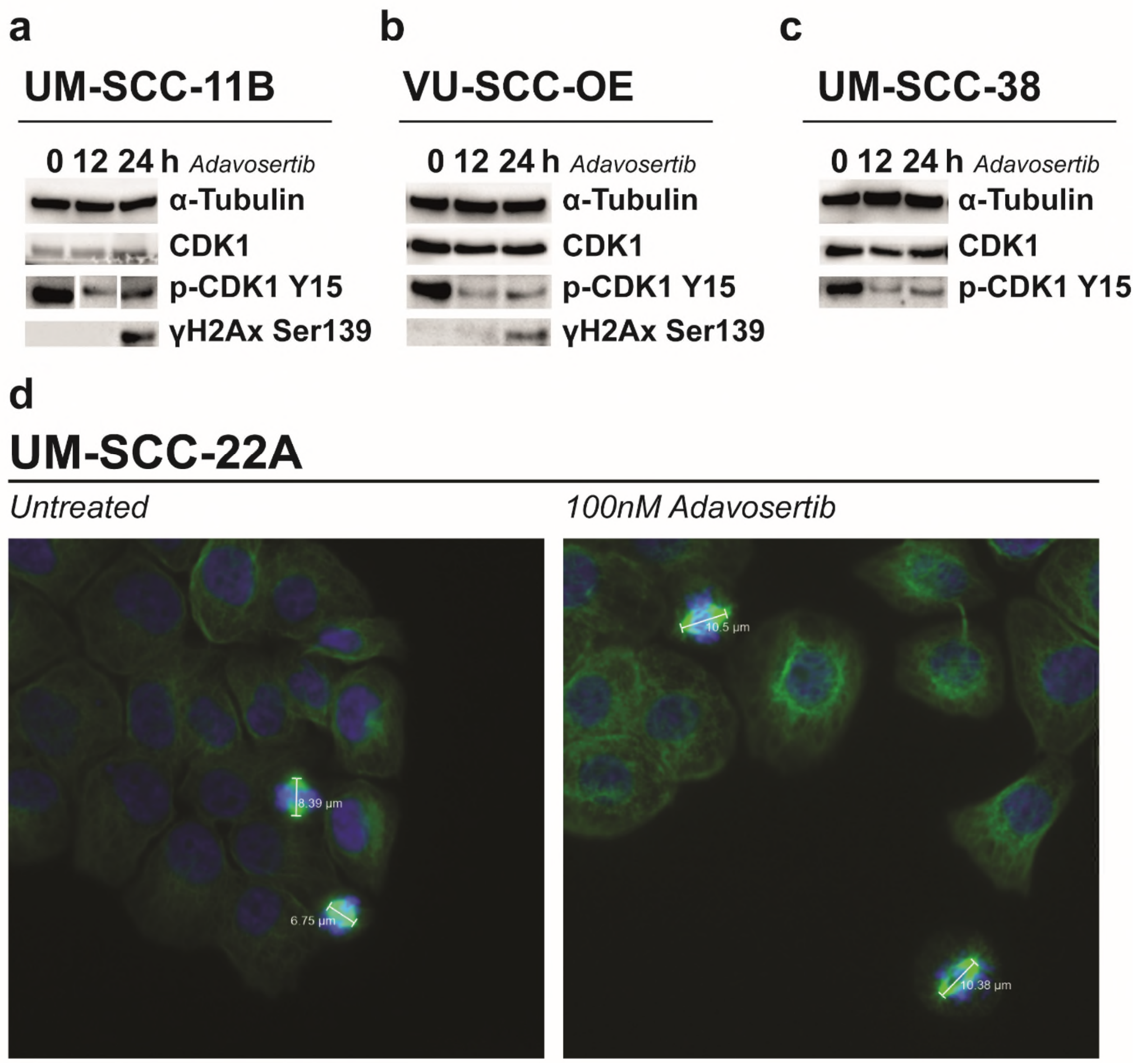

van Harten, de Boer *et al.*

Supplementary figure S5

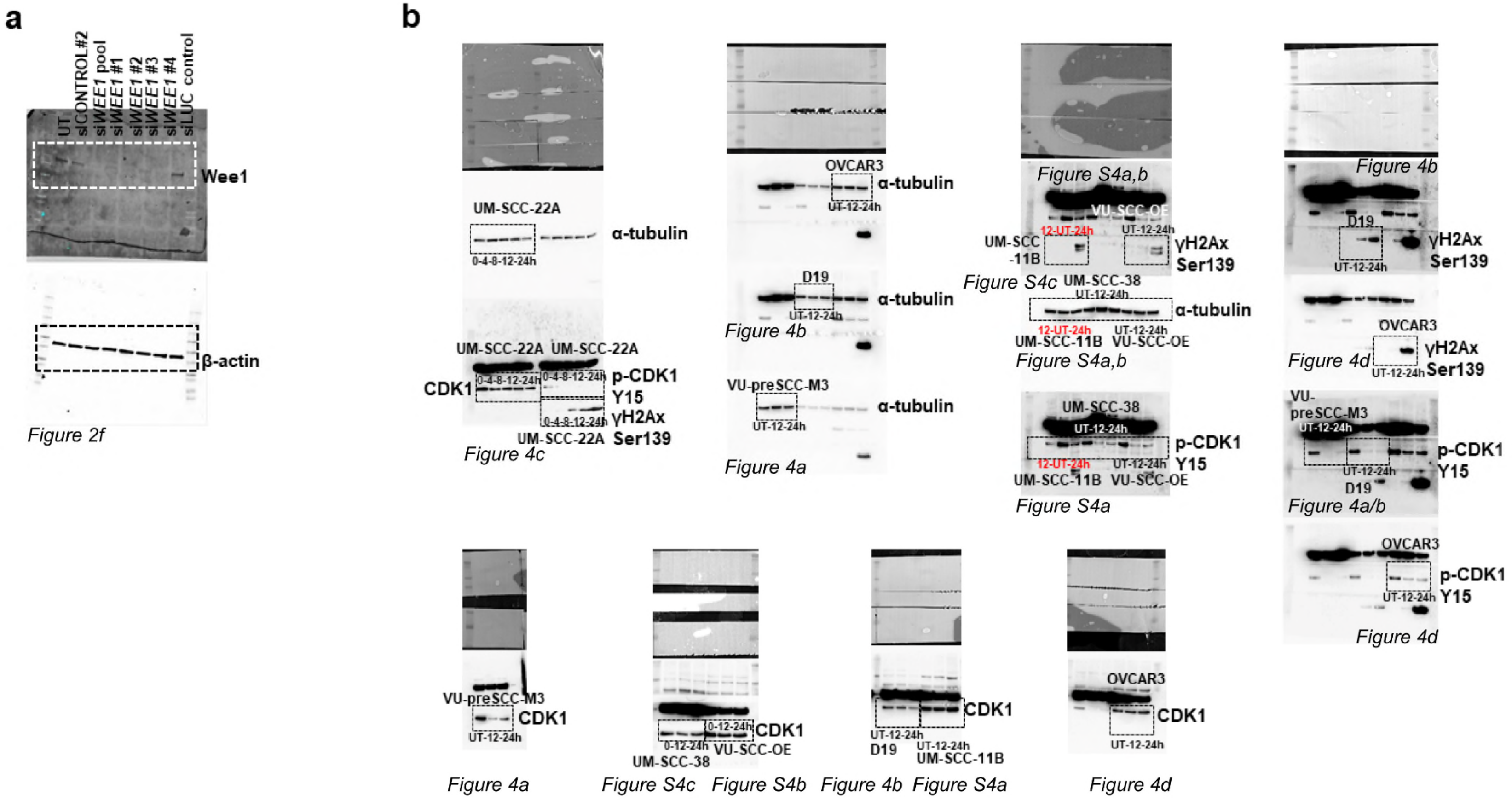

Supplementary table S1: Materials and methods siRNA and Western blot antibodies

	Item	Supplier	Cat. No.
siRNAs	**siCONTOL #2**	GE Healthcare Dharmacon	D-001206-14
	**si*WEE1* #1**	GE Healthcare Dharmacon	D-005050-01
	**si*WEE1* #2**	GE Healthcare Dharmacon	D-005050-02
	**si*WEE1* #3**	GE Healthcare Dharmacon	D-005050-03
	**si*WEE1* #4**	GE Healthcare Dharmacon	D-005050-22
	**si*HPRT1* pool**	GE Healthcare Dharmacon	D-008735-01, -20, -21, -22
	**si*LUC***	Quiet Therapeutics	*N/A*
Antibodies	**Wee1 (D10D2)**	Cell Signaling	13084
	**CDK1**	BD Biosciences	610038
	**p-CDK1 Y15**	Abcam	ab47594
	**α-Tubulin (B-7)**	Santa Cruz	sc5286
	**γH2Ax Ser139**	R&D systems	AF2288
	**β-Actin (N-21)**	Santa Cruz	sc130656
	**IRDye 680RD Goat-anti-Mouse**	Li-COR	926-68070
	**IRDye 800CW Goat-anti-Rabbit**	Li-COR	926-32211

N/A not applicable

Supplementary table S4: *TP53* mutations in cell lines and primary mucosal controls

	Cell line	*TP53* mutation (database R12)	Protein
HPV-negative HNSCC	**UM-SCC-22A**	g.13419 A>G	p.Y220C
		g.14754+1 G>T	N/A
	**FaDu**	g.14070 G>T	p.R248L
	**UM-SCC-14C**	g.14499_14528del	p.C277_E287delinsX
		g.14509 A>T	p.R280S
	**UM-SCC-11B**	g.14052 G>C	p.C242S
	**VU-SCC-120**	g.13160/13161 GC>TT	p.A161F
		g.13206 G>A	p.C176Y
	**VU-SCC-OE**	g.11727_14754del	p.M1_Q331del
	**VU-SCC-096**	g.13338 A>T	p.H193L
	**UM-SCC-38**	g.13075 G>T	p.K132N
Premalignant HN	**D4**	g.13168 C>A	p.Y163X
	**D19**	g.12253 G>T	p.R110L
		g.13167 A>G	p.Y163C
	**D35**	g.13362 ins T	p.L201fs
	**VU-preSCC-M3**	g.13117 G>A	p.W146X
P.O.	**Primary oral keratinocytes**	wild type *TP53*	wild type p53
	**Primary oral fibroblasts**	wild type *TP53*	wild type p53
Ovarian	**SKOV3**	g.12191delC	p.S90fs
	**OVCAR3**	g.14070 G>A	p.R248Q
